# Decrease in quality of life predicts mortality in adult patients with pulmonary arterial hypertension due to congenital heart disease

**DOI:** 10.1007/s12471-015-0666-9

**Published:** 2015-03-04

**Authors:** I.M. Blok, A.C.M.J. van Riel, M.J. Schuuring, M.G. Duffels, J.C. Vis, A.P.J. van Dijk, E.S. Hoendermis, B.J.M. Mulder, B.J. Bouma

**Affiliations:** 1 Department of Cardiology, Academic Medical Centre, University of Amsterdam, Meibergdreef 9, 1105 AZ Amsterdam, The Netherlands; 2 ICIN, Netherlands Heart Institute, Utrecht, The Netherlands; 3 Department of Cardiology, Radboud University Medical Centre, Nijmegen, The Netherlands; 4 Department of Cardiology, University Medical Centre Groningen, Groningen, The Netherlands

**Keywords:** Congenital heart defect, Pulmonary arterial hypertension, Quality of life, Serial, Mortality

## Abstract

**Background:**

Decrease in quality of life (QoL) in left-sided heart failure precedes poor survival, which can be reversed with exercise training. We investigated whether QoL is associated with mortality in pulmonary arterial hypertension due to congenital heart disease (PAH-CHD) patients.

**Methods:**

In this observational study, PAH-CHD adults referred for PAH-specific therapy were included. QoL surveys (SF36) were recorded during 2 years of therapy. Based on shift in SF36 scores during this period, patients had either decreased or non-decreased QoL. Subsequently, the patients were followed for mortality.

**Results:**

Thirty-nine PAH-CHD patients (mean age 42, 44 % male, 49 % Down’s syndrome) were analysed. Following PAH-specific therapy, SF36 physical component summary (PCS) decreased in 13 (35–31 points, *p* = 0.001) and showed no decrease in 26 patients (34–43 points, mean values, *p* < 0.001). Post-initiation phase, median follow-up was 4.5 years, during which 12 deaths occurred (31 %), 10 (56 %) in the decreased and 2 (10 %) in the non-decreased group (*p* = 0.002). Cox regression showed a decrease in SF36 PCS predicted mortality (HR 3.4, 95 % CI 1.03–11, *p* = 0.045).

**Conclusions:**

In PAH-CHD patients, decrease in SF36 PCS following initiation of PAH-specific therapy is a determinant of mortality.

**Electronic supplementary material:**

The online version of this article (doi:10.1007/s12471-015-0666-9) contains supplementary material, which is available to authorized users.

**What is already known about this subject?**

Patients with pulmonary arterial hypertension due to congenital heart disease (PAH-CHD) exhibit high morbidity and mortality. PAH-specific therapy has become the cornerstone in the management of these patients due to beneficial effects on symptoms, haemodynamics and quality of life (QoL). In patients with left-sided heart failure a decrease in QoL is associated with higher mortality, but exercise training in these patients improves both QoL and mortality. It is currently unclear whether these findings are applicable to the PAH-CHD population, and whether QoL monitoring in these patients can identify those with a poor prognosis. Therefore, we investigated whether a decrease in QoL in adult PAH-CHD patients is associated with late mortality.

**What does this study add?**

Our study is the first to report that decrease in QoL is a predictor for late mortality in PAH-CHD.

**How might this impact on clinical practice?**

We recommend periodical assessment of QoL in PAH-CHD patients to facilitate early recognition of disease progression.

## Introduction

Adults with congenital heart disease (CHD) have a lower life expectancy than their healthy counterparts [1], especially when pulmonary arterial hypertension (PAH) develops [2–7]. PAH-specific therapy has become the cornerstone in the management of these patients because of its beneficial effects on disease symptoms, haemodynamics and quality of life (QoL) [3, 8–10]. However, life expectancy and QoL still remain markedly impaired.

In patients with acquired heart failure, several studies found an association between baseline QoL and mortality [11, 12]. In addition, a decrease in QoL over time was significantly associated with increased mortality in these patients [13], suggesting QoL may identify patients in need for interventions to improve their survival. Furthermore, several studies in acquired heart failure revealed that survival and QoL can be improved by exercise training [14–16]. Thus, QoL has the potential to serve as both an important determinant for mortality and a therapeutic target for treatment. The current study aims to investigate whether a decrease in QoL in adult PAH-CHD patients is associated with late mortality.

## Methods

### Study population

This was a prospective multicentre observational study of adult patients with PAH associated with CHD, including patients with Down’s syndrome [17, 18], referred for PAH-specific therapy between January 2005 and May 2013. The current QoL study was part of an ongoing non-randomised trial on PAH-specific therapy in PAH-CHD patients [5]. Patients with all kinds of structural heart defects, including closed defects, could be enrolled. PAH was defined upon the echocardiographic PAH probability (tricuspid regurgitation velocity ≥ 2.9 m/s).

### Study protocol

In the initiation phase of the study, PAH-specific therapy was started according to a standardised protocol [5]. This included the conduction of QoL questionnaires at standardised time intervals. After the initiation phase those QoL questionnaires were used to form individual QoL regression lines in order to divide patients into either a decreased or non-decreased QoL group. Relevant improvement or decrease of QoL was defined as more than two points change during the initiation phase. Baseline (T0) was defined as the start of follow-up to determine survival, which was 2 years after the initiation phase in each patient.

According to the treatment protocol, patients were evaluated with clinical examination, determination of functional class, 6-min walk distance (6-MWD), echocardiography, laboratory tests (including N-terminal pro-brain natriuretic peptide (NT-pro-BNP)) and quality of life questionnaires. These tests were all conducted 24, 21, 18, 15 and 12 months before T0 and at T0, apart from echocardiography which was performed twice a year.

Quality of life was evaluated using the 36-Item Short Form Health Survey (SF36), a well-documented, widely used and validated, self-administered QoL scoring system. It incorporated both a physical and mental component summary (PCS and MCS), thus reflecting physical and mental health. The QoL questionnaires were filled out by the patient or, in case of Down’s syndrome, the patient’s parent or guardian. Survival and in case of death, time and causes of death during follow-up were obtained from the participating hospitals until March 2014. Doppler echocardiography (VIVID 7 General Electric, USA) was performed to evaluate right ventricular function, using tricuspid annular plane systolic excursion (TAPSE) from M-mode recordings. Systolic pulmonary artery pressure was obtained from Doppler recording using the modified Bernoulli equation.

### Statistical analysis

Descriptive data were presented as mean ± SD if normally distributed or median with IQR, as appropriate. Categorical data were evaluated using the chi-square statistic. The change of continuous variables was evaluated using a two-tailed paired *t* test. Independent sample *t* test or Mann-Whitney U test was used for comparison of continuous variables between two groups. Spearman’s analysis was used for correlation of numerous variables, as appropriate. Time to event analysis was performed with Kaplan-Meier estimates of survival. Log-rank test was performed to determine significant differences in mortality rate between the two groups. The associations between predictors and mortality were evaluated using univariate Cox regression analysis. All reported *p* values are two-sided, and values of *p* < 0.05 were considered significant. Statistical analysis was performed with SPSS 20.0 (IBM Corp, Armonk, NY). Since this study focused on shift in QoL, only data from patients who returned more than one questionnaire were used in the final analysis.

## Results

### Patient cohort

**Fig. 1:**
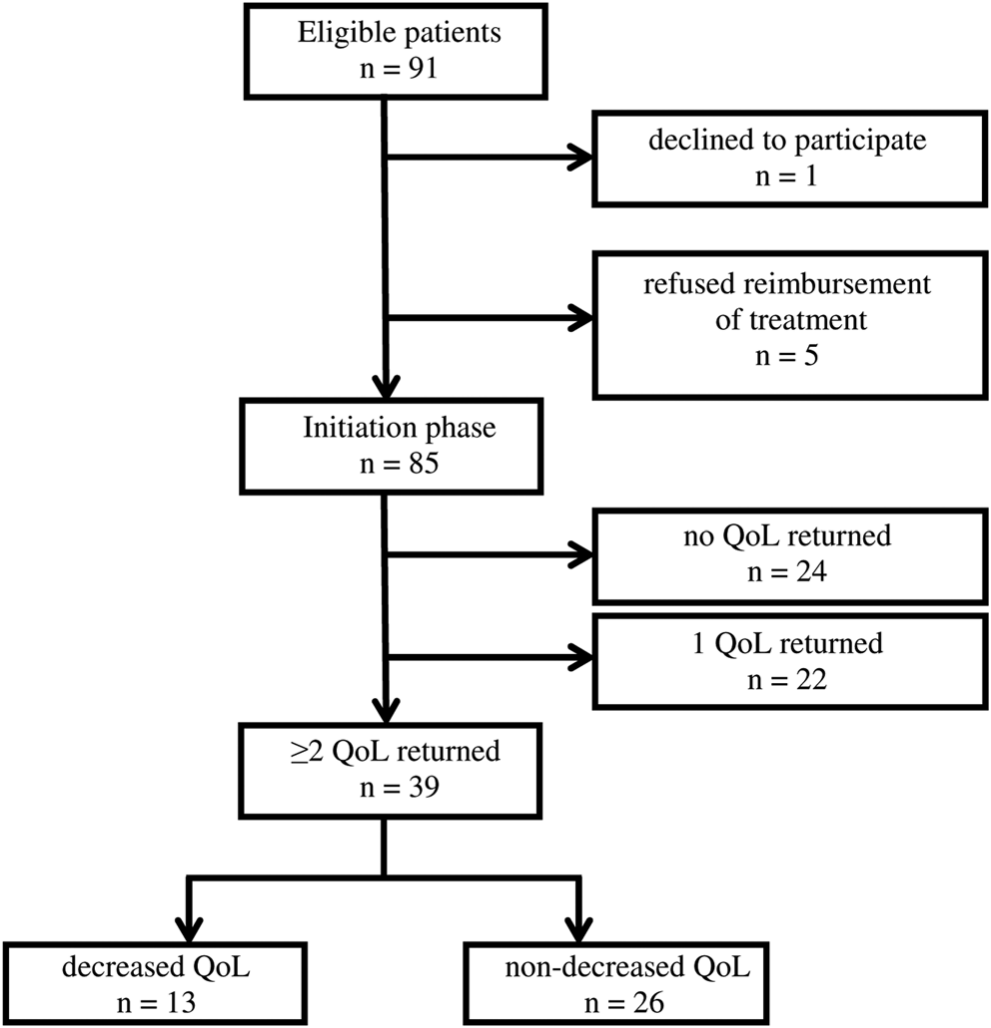
Flowchart of patient inclusion

Ninety-one adults (42 ± 14 year) with PAH-CHD were included (Fig. 1), divided over Amsterdam (64), Nijmegen (15) and Groningen (12). One patient refused treatment initiation. In five patients health insurance refused reimbursement of treatment. In total 85 patients started standardised PAH-specific therapy (initiation phase). Of these, 61 (72 %) patients returned the first QoL questionnaire. Baseline characteristics of these 61 patients are described in the supplementary data (Table S1). In the 61 patients returning the first QoL questionnaire, no differences in QoL were found among those who continued and those who were lost to follow-up, for either the SF36 PCS (mean values 35–35 respectively, *p* = 0.930) or SF36 MCS (mean values 50–48 respectively, *p* = 0.426). In total 39 PAH-CHD patients (mean age 42 ± 12, 44 % male, 49 % Down’s syndrome) completed the initiation phase and their data were used for the final analysis.

**Fig. 2:**
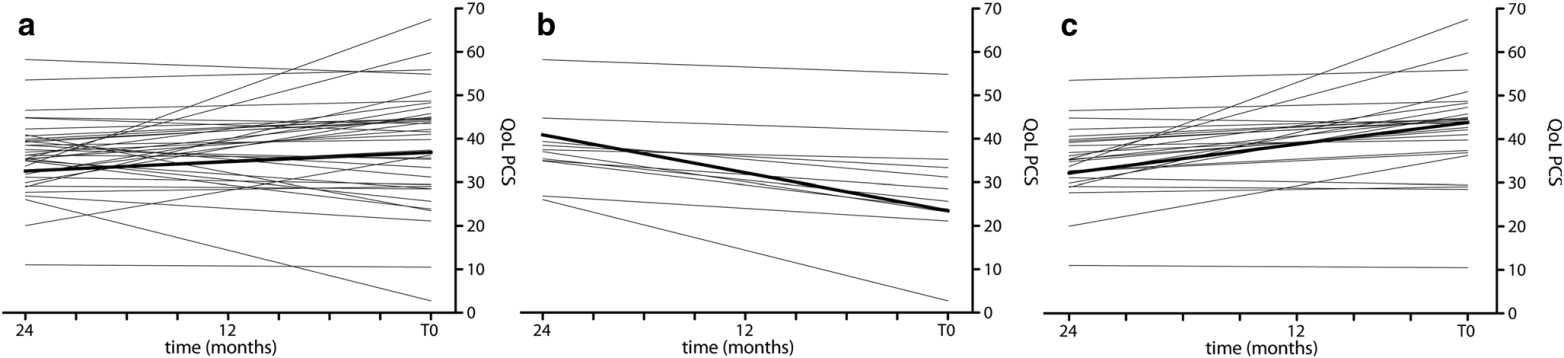
**a** Individual SF36 PCS regression lines of 39 PAH-CHD patients. **b** Decreased SF36 PCS regression lines, 13 patients. **c** Non-decreased SF36 PCS regression lines, 26 patients. All QoL measurements were recorded in the initiation phase after the start of PAH-specific standardised therapy. The thick lines represent the mean regression lines

Baseline characteristics at T0 are summarised in Table 1. Based on their individual SF36 PCS regression lines during the two-year initiation phase (Fig. 2a), patients were divided into two groups. Group 1 consisted of patients with decreased (Fig. 2b) and group 2 of patients without decreased SF36 PCS regression lines (Fig. 2c). At T0 mean SF36 PCS was 38 ± 12 (range 10.96–58.32, *n* = 39), which is more than one SD lower than the general population. SF36 MCS was 49 ± 13 (22.96–66.79, *n* = 39), which closely resembles the mean score of the general population. The two groups, based on either decreased or non-decreased SF36 PCS, were well balanced with respect to most clinical characteristics. However, patients with decreased SF36 PCS had a lower baseline 6-MWD (*p* = 0.009) and New York Heart Association (NYHA) class (*p* = 0.006), compared with patients without a decrease. In contrast, no relation was found between change in SF36 PCS and change in 6-MWD (*r* = 0.12, *p* = 0.468).

**Table 1:** Baseline characteristics at T0

Variables	All patients	Group 1 (decreased QoL)	Group 2 (non-decreased QoL)	*p*
Number	39	13	26	
Age, mean ± SD (years)	42 ± 12	43 ± 10	42 ± 13	0.929
Male, *n* (%)	17 (44)	7 (54)	10 (39)	0.361
Down’s syndrome, *n* (%)	19 (49)	8 (62)	11 (42)	0.257
Eisenmenger’s syndrome, *n* (%)	35 (90)	13 (100)	22 (85)	0.135
Body mass index, mean ± SD (kg)	24 ± 4	25 ± 3	24 ± 4	0.731
NYHA class III or IV, *n* (%)	15 (43)	9 (75)	6 (26)	0.006
SF36 PCS, mean ± SD	38 ± 12	31 ± 11	41 ± 11	0.010
SF36 MCS, mean ± SD	49 ± 13	48 ± 13	50 ± 13	0.716
Medication				
Bosentan, *n* (%)	36 (92)	13 (100)	23 (89)	0.202
Diuretics, *n* (%)	15 (39)	5 (39)	10 (39)	1.000
Cardiac surgery, n (%)	8 (23)	2 (17)	6 (26)	0.529
Age surgery, median (range) (years)	6 (1–66)	5 (2–7)	8 (1–66)	0.643
Saturation in rest, mean ± SD (%)	85 ± 6	86 ± 6	85 ± 7	0.651
NT-pro-BNP, median (IQR) (ng/l)	429 (189–1197)	508 (270–1011)	382 (172–1263)	0.353
6-MWD, mean ± SD, (m)	385 ± 129	311 ± 112	422 ± 123	0.009
TAPSE, mean ± SD, mm	19 ± 6	19 ± 7	19 ± 5	0.785
SPAP, mean ± SD (mmHg)	93 ± 17	98 ± 17	90 ± 17	0.212
Shunt type				
Pre tricuspid, *n* (%)	6 (15)	2 (15)	4 (15)	
Post tricuspid, *n* (%)	18 (46)	5 (39)	13 (50)	0.760
Complex^a^, *n* (%)	15 (39)	6 (46)	9 (35)	

*SF36 PCS* short form health survey of physical health; *SF36 MCS* short form health survey of mental health; *SD* standard deviation; *IQR* interquartile range; *QoL* quality of life; *6-MWD* six-minute walk distance; *SPAP* systolic pulmonary artery pressure; *TAPSE* tricuspid annular plane systolic excursion; *NT-pro-BNP* N-terminal pro-brain natriuretic peptide; *NYHA functional class* New York Heart Association functional class

^a^Complex shunts included atrioventricular septal defect and univentricular heart

**Fig. 3:**
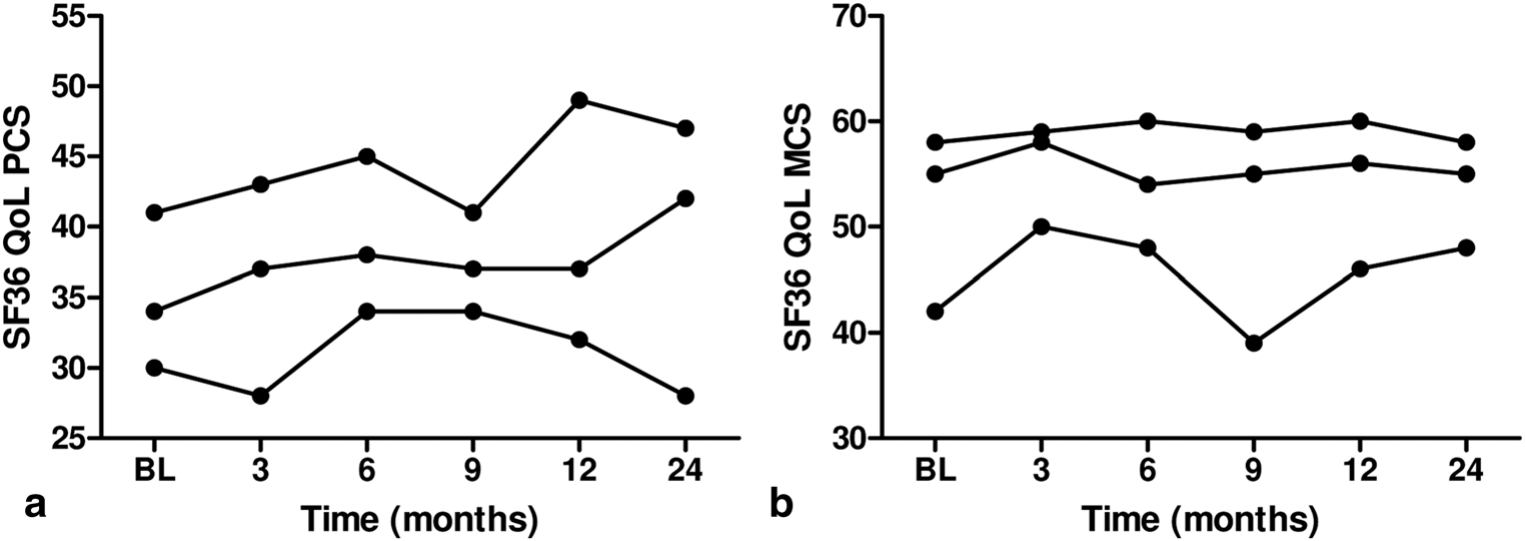
Variability plot showing median, 25 and 75 % values of QoL scores during the initiation phase. **a** Variability of SF36 physical component summary scores. **b** Variability of SF36 mental component summary scores

The 39 patients who finished the initiation phase completed 131 questionnaires (median 4, range 2–5). In 13 patents the SF36 PCS decreased by a mean of ten points (mean values 36–26, *p* = 0.006). In the 26 patients without a decrease, QoL improved in 20 patients (mean values 36–47, *p* = 0.006) and remained stable in 6 (mean values 31–33, *p* = 0.344). Decrease in SF36 PCS was equally distributed in patients with (53 %) and without (40 %) Down’s syndrome (*p* = 0.429). The effect size of decrease was similar in both groups (mean differences 5.3 and 5.1, respectively). Figure 3 shows the variability of the QoL scores during the initiation phase.

### Mortality

During a median follow-up of 4.5 (range 0.3–6.4) years, total all-cause mortality was 31 % (12 deaths). Median time until death was 2.3 years. No patients died during the two-year initiation phase. Two (10 %) patients died in the non-decreased and ten (56 %) in the decreased QoL group (*p* = 0.002). Causes of death were right-sided heart failure (*n* = 7), sudden cardiac death (*n* = 2), sepsis (*n* = 1) and unknown (*n* = 2). Five-year mortality for all patients was 18 %. Seven out of 19 patients with Down’s syndrome died during follow-up compared with 5 out of 20 non-Down patients (*p* = 0.423).

**Fig. 4:**
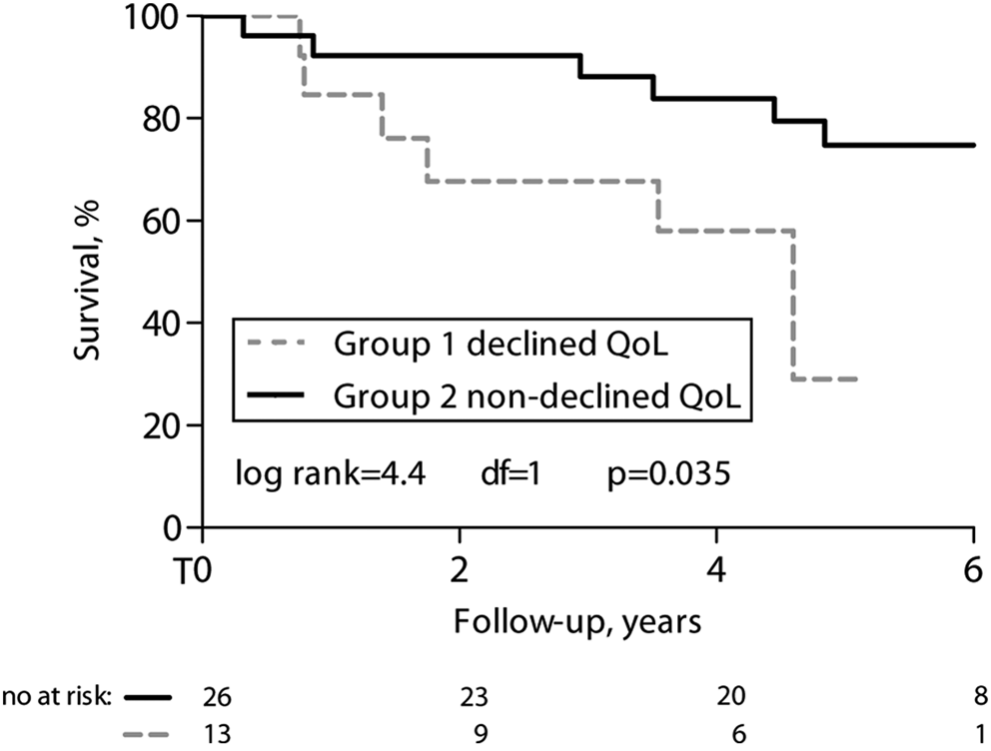
Kaplan-Meier survival curve between PAH-CHD patients with decreased and non-decreased SF36 PCS, 2 years after initiation of PAH-specific therapy

Using univariate Cox regression analysis, the significant determinants of mortality were age, NYHA classification, NT-pro-BNP, 6-MWD and decrease in SF36 PCS (Table 2). Patients with a decrease in SF36 PCS had a 5-year survival of 29 % compared with 74 % in those without a decrease in SF36 PCS (Fig. 4).

**Table 2:** Determinants of survival at T0 in 39 PAH-CHD patients

	Univariate analysis		
	HR	95 % CI	*p*
Male gender	1.11	0.36–3.44	0.863
Age, per 5 years	1.29	1.06–1.56	0.009
Saturation at rest	1.02	0.94–1.12	0.594
Ferritin (µg/L)	0.99	0.97–1.02	0.613
NYHA 3 or 4	4.50	1.30–15.56	0.018
NT-pro-BNP, per 500 ng/L	1.15	1.04–1.27	0.005
6-MWD, per 10 m	0.92	0.87–0.97	0.003
SPAP (mmHg)	1.01	0.97–1.04	0.673
TAPSE (mm)	0.89	0.78–1.02	0.097
QoL PCS	0.98	0.91–1.05	0.534
QoL MCS	1.01	0.95–1.07	0.768
Decreasing saturation at rest	0.93	0.28–3.05	0.898
Decreasing NT-pro-BNP	0.91	0.27–3.04	0.874
Decreasing NYHA class	0.45	0.10–2.06	0.304
Decreasing 6-MWD	2.78	0.85–9.16	0.092
Decreasing SPAP	0.68	0.22–2.15	0.511
Decreasing TAPSE	2.82	0.75–10.64	0.126
Decreasing QoL PCS	3.38	1.03–11.12	0.045
Decreasing QoL MCS	1.61	0.43–5.95	0.478

*HR* hazard ratio; *QoL* quality of life; *PCS* physical summary score; *MCS* mental summary score; *6-MWD* 6-min walk distance; *SPAP* systolic pulmonary artery pressure; *TAPSE* tricuspid annular plane systolic excursion; *NYHA functional class* New York Heart Association functional class; *NT-pro-BNP* N-terminal pro-brain natriuretic peptide

## Discussion

### Quality of life and mortality

The current study shows, for the first time, that a decrease in QoL on the physical component of the SF36 during the initiation phase of PAH-specific therapy in adult PAH-CHD patients is associated with late mortality. Patients with a decrease in SF36 PCS had a 5-year survival of 29 % compared with 74 % in those without a decrease in SF36 PCS. Our finding is in line with previous studies demonstrating an association between QoL and mortality, in healthy people [19] and in patients with acquired heart disease [11, 12, 20]. The above-mentioned studies used single instead of serial QoL measurements. In 2013, Apers et al. proposed that serial follow-up on QoL could be of more value than single measurements [21]. Four studies examined QoL with serial follow-up in CHD. They identified psychosocial determinants of baseline and serial QoL [22, 23], analysed associations between QoL and exercise capacity over time [24] and provided insight into the longitudinal course of QoL [25]. None of these studies evaluated mortality. We, however, demonstrate that assessing QoL with serial follow-up provides useful information about late mortality, suggesting that serial follow-up may be a robust guide to clinical management in CHD patients. The use of patient-reported outcomes such as QoL instruments in clinical practice could help to screen for functional problems, to monitor disease progression or treatment effect, to stratify patients according to mortality risk and to guide clinical management.

In order to implement serial QoL follow-up in clinical practice, its predictive value for mortality has to be weighed against traditional determinants of mortality in PAH-CHD. Unfortunately, multivariate regression analysis could not be performed in our study due to the small number of events. However, despite our number of events, decrease in QoL was significantly associated with mortality, making it a promising determinant.

It is possible that physical quality of life is a proxy for disease severity. In accordance, patients with decreased QoL on the physical component of the SF36 in our study had a lower 6-MWD and worse NYHA class implying more advanced disease. However, if decrease in QoL were merely a proxy for disease severity, one would expect baseline SF36 PCS to predict mortality as well and this was not the case. Given the results described above, we suggest the use of serial QoL follow-up in clinical practice to determine a patient’s mortality.

The relationship between decrease in QoL and late mortality in adult PAH-CHD patients, as shown in our study, emphasises the importance of screening for decrease in QoL since periodical assessment could facilitate an early recognition of disease progression. A critical question is whether survival can be optimised in patients with disease progression. The use of knowledge from other areas of heart disease can be important for this task. In patients with acquired heart failure, baseline QoL was found to be a reliable determinant for mortality [11, 12]. Additionally a study by Lupon et al. in 1151 heart failure patients focused on shift in QoL [13]. They used Minnesota QoL questionnaires for up to 5 years with extended follow-up to 6 years for prognosis assessment. During this period 457 deaths were recorded. A decrease in QoL (score deterioration of ≥ 10 %) was associated with impaired 12-month survival (HR 1.012, 95 % CI 1.006–1.018, *p* < 0.001). On top of QoL serving as a determinant for mortality, three other studies reported that stimulation of physical activity has shown to be effective for improving QoL, as well as mortality [14–16]. It might be interesting for future studies in PAH-CHD patients to evaluate whether an improvement of QoL influences survival.

### Study limitations

Quality of life questionnaires have not been properly validated in patients with Down’s syndrome. To address this problem and increase their validity, QoL questionnaires were filled in by the patient’s parent or guardian. Since self-reports are impossible in these patients, even though the SF36 has not been validated for proxy report, it is the most appropriate way to evaluate QoL in Down’s syndrome patients. This approach has yielded favourable results in earlier studies [3, 5, 26] and makes sure new scientific insights are also gained for patients with Down’s syndrome. In this study, decrease in SF36 PCS was equally distributed and had a similar effect size for both Down and non-Down patients. However, further research is crucial to determine the value of questionnaires by proxy reports.

Although our study comprises the largest Dutch cohort of PAH-CHD patients, we were unfortunately unable to perform multivariate regression analysis due to the number of events.

Finally, invasive haemodynamics are recommended in the guidelines for the diagnosis of PAH [27, 28]. However, echocardiography is an adequate non-invasive modality in patients with evident diagnosis of PAH [29, 30] in patients with CHD. Due to the higher complication risk in patients with PAH-CHD cardiac catheterisation was not routinely performed in these patients, but only if the diagnosis of PAH was not clearly evident at echocardiography.

## Conclusion

A decrease in QoL on the physical component of the SF36 after the initiation of PAH-specific therapy in adult PAH-CHD patients predicts mortality. Patients with a decrease in SF36 PCS during the initiation phase have a 5-year survival of 29 % compared with 74 % in those without a decrease in SF36 PCS. Periodical assessment of quality of life in PAH-CHD patients could facilitate early recognition of disease progression. Based on the association between QoL and mortality, it might be interesting for future studies in PAH-CHD patients to evaluate whether interventions to improve QoL might influence survival.

## Electronic supplementary material

(PDF 60kb)
