# Supplementary material for: Decrease in quality of life predicts mortality in adult patients with pulmonary arterial hypertension due to congenital heart disease
Source: Neth Heart J. 2015 Mar 4;23(5):278–84. doi: 10.1007/s12471-015-0666-9 (PMC4409603; doi:10.1007/s12471-015-0666-9)
Supplement: Supplementary file 1 — (PDF 60kb) [file 12471_2015_666_MOESM1_ESM.pdf]

SUPPLEMENTARY DATA

Table S1. Baseline Characteristics at start standardized PAH-specific treatment protocol

Variables	*All Patients*	*1 QoL questionnaire*	*>1 QoL questionnaire*	*p*
Number	61	22	39	
Age, mean ± SD, y	49 ± 13	52 ± 15	47 ± 12	0.047
Male, n (%)	26 (43)	9 (41)	17 (44)	0.839
Down syndrome, n (%)	23 (38)	4 (18)	19 (49)	0.018
Eisenmenger syndrome, n (%)	48 (79)	13 (59)	35 (90)	0.005
Body Mass index, mean ± SD, kg	24 ± 5	25 ± 6	24 ± 5	0.494
NYHA class III or IV, n (%)	31 (51)	16 (73)	15 (39)	0.025
SF36 PCS, mean ± SD	35 ± 9	35 ± 11	35 ± 8	0.930
SF36 MCS, mean ± SD	49 ± 12	48 ± 13	50 ± 11	0.426
Medication				
-bosentan, n (%)	61 (100)	22 (100)	39 (100)	1.000
-diuretics, n (%)	22 (39)	7 (39)	15 (39)	0.975
Cardiac surgery, n (%)	17 (28)	9 (41)	8 (21)	0.006
-Age surgery, median (range), y	4 (1-75)	4 (2-75)	6 (1-66)	0.867
Saturation in rest, median (IQR), %	87 (80-93)	88 (80-96)	85 (80-91)	0.256
NT-pro-BNP, median (IQR), ng/L	466 (218-1209)	424 (161-1623)	556 (244-1127)	0.500
6-MWD, mean ± SD, m	377 ± 124	401 ± 125	364 ± 123	0.279
TAPSE, median (IQR), mm	19 (16-23)	17 (15-22)	19 (16-23)	0.293
SPAP, median (IQR), mmHg	89 (71-98)	81 (49-91)	91 (76-103)	0.016
Shunt Type				
-pre tricuspid, n (%)	11 (18)	5 (23)	6 (15)	
-post tricuspid, n (%)	27 (44)	9 (41)	18 (46)	0.769
-complex*, n (%)	23 (38)	8 (36)	15 (39)	

Abbreviations: SF36 PCS, short form health survey of physical health; SF36 MCS, short form health survey of mental health; SD, standard deviation; IQR, interquartile range; QoL, quality of life; 6-MWD, six-minute walk distance; SPAP, systolic pulmonary artery pressure; TAPSE, tricuspid annular plane systolic excursion; NT-pro-BNP, N-terminal pro-brain natriuretic peptide; NYHA functional class, New York Heart Association functional class.

* Complex shunts included atrioventricular septal defect and univentricular heart.
